# Savings and extinction of conditioned eyeblink responses in fragile X syndrome

**DOI:** 10.1111/j.1601-183X.2008.00417.x

**Published:** 2008-10

**Authors:** A E Smit, J N van der Geest, M Vellema, S K E Koekkoek, R Willemsen, L C P Govaerts, B A Oostra, C I De Zeeuw, F VanderWerf

**Affiliations:** † Department of NeuroscienceErasmus MC, Rotterdam, The Netherlands; ‡ Bio-Imaging Lab, Department of Biomedical Sciences, University of AntwerpAntwerp, Belgium; § Department of Behavioural Neurobiology, Max Planck Institute for OrnithologySeewiesen, Germany; ¶ Department of Clinical Genetics, Erasmus MCRotterdam, The Netherlands; †† Netherlands Institute of NeuroscienceAmsterdam, The Netherlands

**Keywords:** Cerebellum, delay eyeblink conditioning, extinction, fragile X syndrome, human, savings

## Abstract

The fragile X syndrome (FRAXA) is the most widespread heritable form of mental retardation caused by the lack of expression of the fragile X mental retardation protein (FMRP). This lack has been related to deficits in cerebellum-mediated acquisition of conditioned eyelid responses in individuals with FRAXA. In the present behavioral study, long-term effects of deficiency of FMRP were investigated by examining the acquisition, savings and extinction of delay eyeblink conditioning in male individuals with FRAXA. In the acquisition experiment, subjects with FRAXA displayed a significantly poor performance compared with controls. In the savings experiment performed at least 6 months later, subjects with FRAXA and controls showed similar levels of savings of conditioned responses. Subsequently, extinction was faster in subjects with FRAXA than in controls. These findings confirm that absence of the FMRP affects cerebellar motor learning. The normal performance in the savings experiment and aberrant performance in the acquisition and extinction experiments of individuals with FRAXA suggest that different mechanisms underlie acquisition, savings and extinction of cerebellar motor learning.

With an estimated prevalence of 1 in 4000 men and 1 in about 6000 women, the fragile X syndrome (FRAXA) is the most widespread heritable form of mental retardation. An elongated facial structure, large protruding ears, hyperextensible joints and macroorchidism characterize individuals with FRAXA. The mental retardation can be accompanied by various degrees of hyperarousal, attention deficits, anxiety, social withdrawal, autistic behavior and seizure susceptibility (Beckel-Mitchener & Greenough 2004; Crawford *et al.* 1999; Jin *et al.* 2004; Turner *et al.* 1996; de Vries *et al.* 1997).

Several studies suggest that the cerebellum is involved in FRAXA (Gothelf *et al.* 2008; Hessl *et al.* 2004; Huber 2006; Koekkoek *et al.* 2005). On a molecular level, the fragile X mental retardation protein (FMRP) is strongly expressed in the cerebellum of healthy individuals, especially in Purkinje cells which comprise the sole output of the cerebellar cortex (Tamanini *et al.* 1997). In FRAXA, hyperexpansion of a trinucleotide CGG repeat in the fragile X mental retardation gene, *FMR1*, results in transcriptional silencing of FMRP (Verkerk *et al.* 1991). On a morphological level, FMRP is a translational suppressor at synaptic sites in dendrites and important for normal spine maturation and pruning (Bagni & Greenough 2005; Comery *et al.* 1997). *FMR1*-null mouse mutants, in which the expression of FMRP is absent, have abnormally shaped spines on dendrites of Purkinje cells. However, the spine density and shape of the dendritic tree appear to be normal (Koekkoek *et al.* 2005). In patients with FRAXA, magnetic resonance imaging revealed hypoplasia of the cerebellar vermis, in particular of lobules VI and VII (Gothelf *et al.* 2008; Hessl *et al.* 2004).

On a behavioral level, patients with FRAXA show aberrant delay eyeblink conditioning (Koekkoek *et al.* 2005). In an eyeblink conditioning test, repeated paired exposure to an air puff on the cornea (US: unconditioned stimulus), which evokes a reflex blink, and a tone (CS: conditioned stimulus), which by itself does not evoke a blink, results in a blink in response to not only the air puff but also the tone alone (CR: conditioned response) (Domjan 2005). Delay eyeblink conditioning, in which the termination of the CS and US coincides, is largely dependent on cerebellar functioning (Bracha 2004; Christian & Thompson 2003, 2005; Woodruff-Pak *et al.* 1996), which is thought to control the precise timing of the CR (Gerwig *et al.* 2005; Koekkoek *et al.* 2003).

Acquisition of the CR is thought to be mediated by long-term depression of the parallel fiber–Purkinje cell synapses induced by concurrent stimulation of the parallel fiber system by the CS (tone) and the climbing fiber system by the US (air puff) (Kitazawa 2002). In a previous study, we observed that in global and Purkinje-cell-specific knockout mice, absence of FMRP leads to enhanced parallel fiber long-term depression (Koekkoek *et al.* 2005). A lack of FMRP suppression might lead to an exaggeration of translation responses linked to group I metabotropic glutamate receptors (mGluRs 1 and 5), which are involved in the consolidation of long-term depression (Bear *et al.* 2004; Vanderklish & Edelman 2005).

Deviant cerebellar functioning could explain the lower performance of FMRP mouse mutants and patients with FRAXA in the short-term acquisition of the delay conditioned eyeblink response (Koekkoek *et al.* 2005). In the present article, we investigated the long-term mechanisms of delay eyeblink conditioning in individuals with FRAXA. Presence of an increased percentage of CRs a long time after CR acquisition (savings) would indicate that normal expression of FMRP in cerebellar Purkinje cells is necessary for learning but not for remembering a conditioned eyeblink response. On the other hand, deviant savings behavior in individuals with FRAXA suggests that both learning and storage of the memory traces in delay eyeblink conditioning are dependent on similar mechanisms. Likewise, FMRP may also be involved in the ability to actively forget the CR (extinction).

Thus in the present study, we investigated savings and extinction behavior of the delay conditioned eyeblink response in individuals with FRAXA to study the long-term effects of disturbed FMRP expression.

## Materials and methods

### Experimental subjects

Written informed consent was obtained from (the parents of) 11 male subjects with FRAXA (21–39 years of age: average 31 ± 6.5 years) and 14 healthy male control subjects (22–45 years of age: average 30 ± 6.0 years). The EramusMC Medical Ethics Committee (MEC-2004-205) approved the protocol. The participants with FRAXA had full mutations as they had more than 200 CGG repeats in their *FMR1* gene (data not shown).

All subjects could distinguish at least six out of ten 650 Hz, 500 millisecond tones presented at different volumes from a continuous 60 dB white noise background, so their hearing was sufficient to participate. During the experiments, all participants displayed reflex blinks in response to an air puff with an onset latency of less than 100 milliseconds, which indicated that they all had normal eyelid motor function. Spontaneous blink rates did not differ between the FRAXA and the control group (6.0 ± 3.3 and 7.6 ± 5.0 blinks/min, respectively, *P* = 0.501).

### Apparatus

All subjects were seated in front of a monitor and watched a movie of their own choice during the experiments. The eyeblink conditioning setup consisted of a headset with an air puff nozzle attached to the right side of the headphone and magnetic distance measurement technique (MDMT) equipment (Koekkoek *et al.* 2002). The headset allowed free head movement and provided sound isolation from the environment. The setup was especially designed to be able to perform experiments with individuals with FRAXA. Free head movement and entertainment with the movie were essential to ensure that attention deficits and anxiety would not interfere with the eyeblink conditioning sessions. The air puff nozzle was directed at the cornea close to the outer canthus of the right eye at a distance of about 15 mm. It served to deliver a 20 millisecond air puff with an intensity of 10–50 pounds per square inch (PSI) at the source (the US: unconditioned stimulus). The intensity could be adjusted to elicit a single blink reflex. The sound of the movie (background noise) and the 650 Hz, 75 dB, 520 millisecond tones (the CS: conditioned stimulus) were presented bilaterally through the headphones. The MDMT equipment was originally designed for mice and is described in detail elsewhere (Koekkoek *et al.* 2002) and was modified for the present study in human subjects. In short, the MDMT consists of a giant magnetoresistive sensor attached to the edge of the lower orbit of the right eyelid. It measures the distance to two small gold-plated neodymium magnets (N43 1.6 × 0.7 × 0.8 cm, with the long side positioned parallel to the eyelid) attached to the edge of the right upper eyelid. The signal was amplified by a preamplifier close to the sensor (×10), and further amplification (between 10–50 times) could be adjusted per subject. The MDMT signal was digitized (1000 Hz) using National Instruments hardware. A custom-made labView (National Instruments, Austin, TX, USA) script controlled the monitoring of eyelid movement, presentation of the stimuli and capturing of data on disk.

### Procedure

Subjects participated in three experiments in which acquisition, savings and extinction of delay eyeblink conditioning were assessed. Eleven subjects with FRAXA and 14 controls participated in the study.

**Figure 1:**
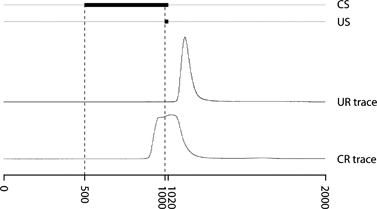
Description of a trial Each trial lasted 2000 milliseconds, during which either an air puff alone (US only), a tone alone (CS only) or a paired CS and US stimulus could be presented. The 520 millisecond tone (CS) would start at 500 milliseconds after trial onset, the 20 millisecond air puff (US) would start at 1000 milliseconds after trial onset and both stimuli would end at 1020 milliseconds in the trial. During each trial, the eyelid movement (trace) is recorded, and blinks are shown as peaks in the recording. Traces of an unconditioned response (UR trace) and a conditioned response (CR trace) are shown. An eyelid response with an onset latency between 650 and 1025 milliseconds with respect to the start of a CS-only or paired trial was considered a conditioned response.

The protocol for the acquisition experiment was similar to that of our previous study (Koekkoek *et al.* 2005). In this experiment, subjects received 10 blocks of eight 2-second trials. Each block consisted of one trial in which the air puff was presented alone (US only), one trial in which the tone was presented alone (CS only) and six trials in which tone and air puff were paired. In these paired trials, the 20 millisecond air puff was presented with a 500-millisecond delay after onset of the 520 millisecond tone, so the two stimuli ended simultaneously (delay eyeblink conditioning, see Fig. 1). The three types of trials (US only, CS only and paired) were presented in random order within each of the 10 blocks. The trials were separated by a random interval of 20–30 seconds, and blocks were separated by 1 min.

The savings experiment was performed at least 6 months after the acquisition experiment (average 9.2 ± 3.4 months) using the same protocol. At the end of the savings experiment, the extinction experiment was performed. In the extinction experiment, each block consisted of 7 CS-only trials, yielding 70 trials in total, again randomly separated by 20–30 seconds.

Eyelid position was constantly monitored with the MDMT signal. Trials were started only when the eyelid was fully open, which was determined automatically using a threshold. This threshold was manually adjusted to above the position that was measured when the eye was open.

### Raven's tests

A rough estimate of nonverbal cognitive performance of the subjects with FRAXA was obtained using Raven's colored progressive matrices in 10 subjects and Raven's standard progressive matrices in 1 subject. Raw scores were transformed into IQ scores (Raven 2000).

### Data analysis

For each CS-only and paired trial, the presence or absence of a conditioned response (CR) was determined semiautomatically using custom written scripts in matlab (version 6.5; The Mathworks, Inc. Natick, MA, USA) and labView (versions 7.1 and 8.2; National Instruments Austin, TX, USA). First, the MDMT data were filtered with a seventh-order Butterworth 100 Hz low-pass filter. The program looked for a maximum eyelid closure between 600 and 1300 milliseconds and calculated the range between this maximum and the absolute minimum between 600 milliseconds and the time of the maximum. Subsequently, the first local minimum before the maximum eyelid closure under 5% of this range was determined. The time-point of this local minimum could be manually adjusted to ensure proper detection and was taken as the onset of the eyeblink.

A trial was considered invalid when the 2-second recording contained more than three peaks or when the maximum eyelid closure during a blink occurred between 0 and 100 milliseconds after CS onset. The latter was considered a startle response to the tone. These trials were discarded from the analysis.

A CR was defined as a blink with an onset latency between 150 milliseconds (650 milliseconds in the trial) and 525 milliseconds (1025 milliseconds in the trial) after the start of the CS, to exclude eyelid reflexes in response to the air puff, which was presented 500 milliseconds after the tone.

For each subject and experiment, we calculated the percentage of CRs within the blocks, taking only valid trials into account. We compared the percentage of CRs between subjects with FRAXA and controls for each of the three experiments as a whole and for each block within an experiment. To assess overall learning, we compared the percentages of CRs from the beginning of the acquisition experiment to the end of the savings experiment. To study savings in particular, we compared the percentage of CRs between the first block of the acquisition experiment and the first block of the savings experiment. To evaluate extinction, we compared the percentage of CRs between the last block of the savings experiment and the last block of the extinction experiment for both the FRAXA and the control group and looked at the differences between the groups. In the latter analysis, only participants with scores more than 50% CRs in the last two blocks of the savings experiment were included to ensure that we studied individuals who did acquire a delay conditioned eyeblink response.

All comparisons were done with the nonparametric Mann–Whitney test using spss (version 11.0; SPSS Inc., Chicago, IL, USA), and the level of significance in all analyses was set at *P* < 0.05. In *Results*, averages and standard deviations are presented.

## Results

**Figure 2:**
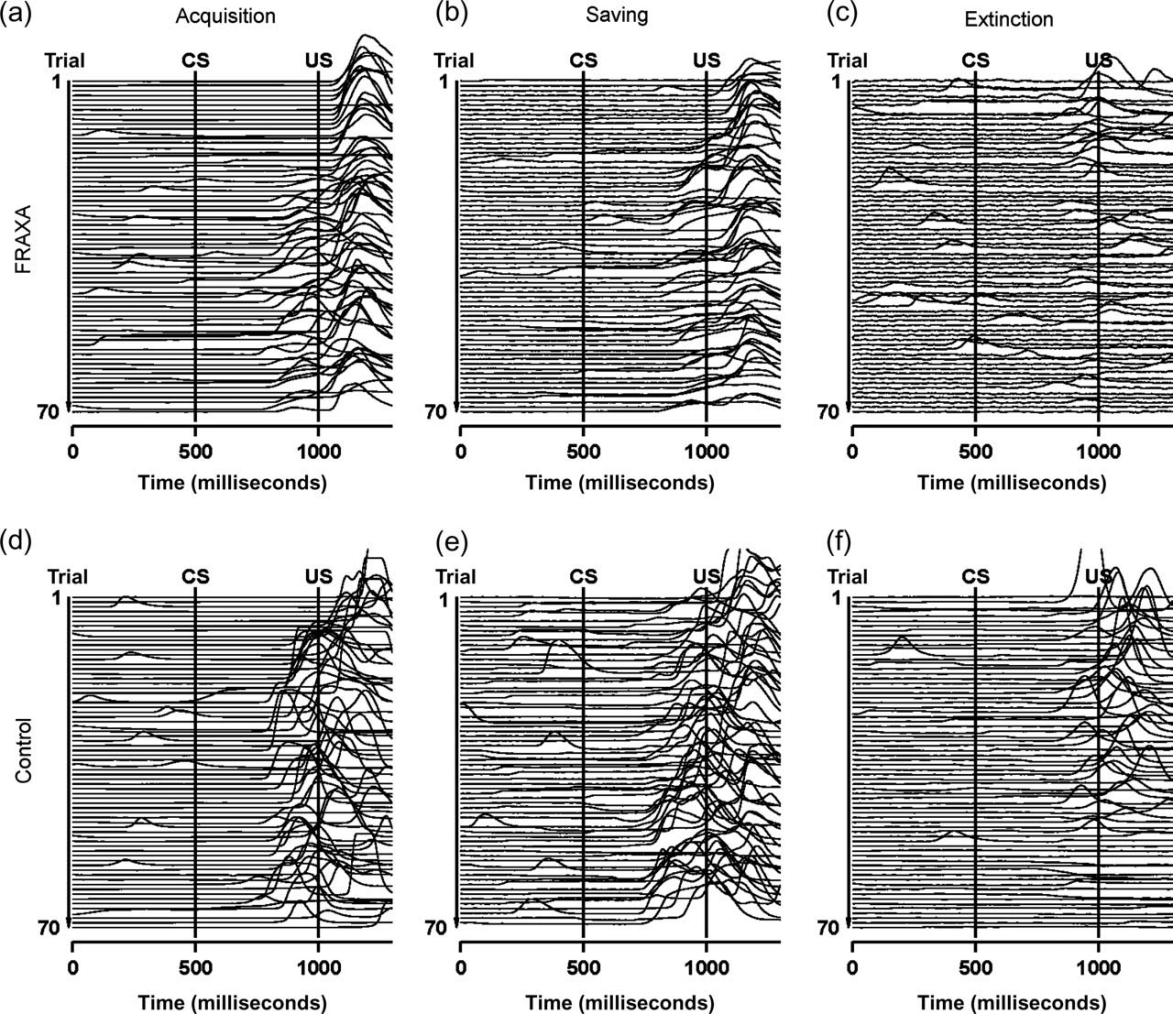
Exemplary raw eyelid movement data of a subject with FRAXA (upper row) and a control subject (lower row) during the acquisition (a, d), savings (b, e) and extinction (c, f) experiments. Recordings of the 70 CS-only and paired trials are shown in order, starting with the first trial at the top. The two vertical lines indicate CS onset (at 500 milliseconds) and US onset (at 1000 milliseconds).

Three experiments of delay eyeblink conditioning were performed to assess (1) acquisition, (2) saving and (3) extinction of a conditioned response (CR) in both male subjects with FRAXA and male controls. Individual traces of eyelid movements of a subject with FRAXA and a control for each of the three experiments are shown in Fig. 2.

### Acquisition of the CR

Both the subject with FRAXA (Fig. 2a) and the control (Fig. 2d) did acquire the CR. However, the forward shift in the onset latency of this response does not start until the fourth block in the subject with FRAXA.

**Figure 3:**
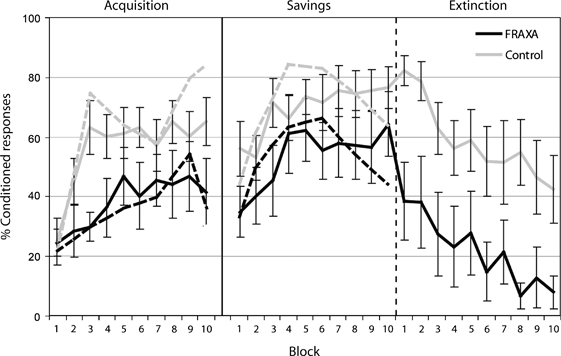
The average percentages of conditioned responses in CS-only and paired trials per block for the FRAXA group (black line) and control group (gray line) for each of the three experiments (acquisition, savings and extinction) are displayed. Error bars represent standard error of the mean. For clarity of presentation, conditioned responses in CS-only trials are displayed as dashed lines without error bars. Both groups showed an increase in conditioned response incidence across the acquisition and savings experiment and a decrease in the extinction experiment.

The learning curves of the FRAXA group and the control group in the first delay eyeblink conditioning experiment are plotted in the first part of Fig. 3. Over the whole experiment, the FRAXA group exhibited less CRs than the control group (38.4 ± 8.2% vs. 56.6 ± 12.6% of the trials, respectively, *P* = 0.106). In the first two blocks, the number of CRs was not different between the two groups, but the number of CRs in the FRAXA group was lower in block 3 (*P* = 0.009) but not significantly different in blocks 4–10. The increase in the percentage of CRs between the first and last blocks of the acquisition experiment was significant in controls (25.0 vs. 67.2, *P* = 0.003) but not in subjects with FRAXA (23.9 vs. 45.5, *P* = 0.501).

The average onset latency of the CRs was similar in subjects with FRAXA (850 ± 45 milliseconds) and controls (856 ± 37 milliseconds, *P* = 0.640).

### Savings of the CR

Eyelid movements measured during the savings experiment of the two exemplary subjects are shown in Fig. 2b (FRAXA) and Fig. 2e (control). Both subjects clearly start displaying CRs earlier than they did in the acquisition experiment, but the subject with FRAXA is slower. He starts showing CRs at the end of the second block, and the control already starts in the first.

The percentages of CRs during the savings experiment after approximately 6 months are plotted in the second part of Fig. 3. In general, the FRAXA group exhibited slightly less CRs than the control group (53.6 ± 9.3% vs. 69.4 ± 8.4% of the trials, respectively, *P* = 0.261), and no significant differences between the two groups were observed for individual blocks as well. The differences in the percentage of CRs between the first block of the savings experiment and the first block of the acquisition experiment were not significant between subjects with FRAXA (33.9 ± 43.5%) and controls (51.5 ± 38.6%, *P* = 0.143).

The average onset latency of CRs in the savings experiment was similar in subjects with FRAXA (860 ± 51 milliseconds) and controls (855 ± 46 milliseconds, *P* = 0.891).

**Figure 4:**
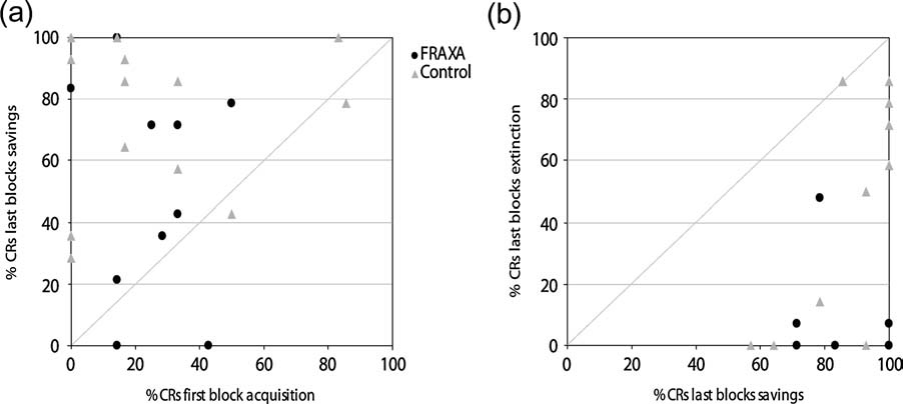
A scatter plot showing the average percentage conditioned responses (CRs) per subject of the first block of acquisition vs. the last two blocks of saving (a) and the last two blocks of savings and the last two blocks of extinction (b). Most subjects with FRAXA (black dots) and most control subjects (gray triangles) show an increased percentage of CRs at the end of the savings experiment compared with the beginning of the acquisition experiment. Two subjects with FRAXA had 14.3% and 100.0% of CRs, and two control subjects had 0.0% and 100.0% of CRs in the first block of acquisition and last two blocks of savings, respectively. Six subjects with FRAXA and 10 controls had more than 50% of CRs in the last two blocks of their savings experiment and were included in the analyses of the extinction experiment. All these subjects display fewer CRs at the end of the extinction experiment compared with the end of the savings experiment.

Repetition of the acquisition protocol induces a significant increase in the percentage of CRs, when we compared the end of the savings experiment with the beginning of the acquisition experiment in both the FRAXA group (24.6 ± 14.8% vs. 58.4 ± 35.8%, *P* = 0.004) and the control group (25.0 ± 29.5% vs. 76.5 ± 26.6%, *P* < 0.001). However, the overall learning of CRs was not different between the two groups (*P* = 0.827; Fig. 4a).

### Extinction of the CR

Eyelid movement traces of the extinction experiment of a subject with FRAXA and a control are shown in Fig. 2. The subject with FRAXA (Fig. 2c) unlearns the CR within three blocks, whereas the control subject (Fig. 2f) exhibits CRs until block 7. Five of the 11 subjects with FRAXA and 4 of the 14 controls did not reach 50% CRs in the last two blocks of the savings experiment. These subjects were discarded from further analysis. The average learning curve of the remaining 6 subjects with FRAXA and 10 controls is plotted in the third part of Fig. 3.

The average percentage of CRs in the whole extinction experiment was lower in the FRAXA group (21.8 ± 11.4%) than in the control group (58.6 ± 13%, *P* = 0.005). The difference between the groups was significant for the first two blocks (*P* = 0.0135, *P* = 0.030) and for blocks 4 and 8 (*P* = 0.024, *P* = 0.005). The difference in percentage of CRs between the last savings block and the last extinction block was not significantly different between the FRAXA group (75.4 ± 29.3%) and the control group (46.2 ± 29.7%, *P* = 0.095; Fig. 4b).

The average onset latency of CRs in the extinction experiment was similar in subjects with FRAXA (830 ± 38 milliseconds) and controls (842 ± 38 milliseconds, *P* = 0.594).

### IQ of the subjects with FRAXA

The IQ scores of the subjects with FRAXA were on average 74 (±6). We observed no significant correlation between IQ and the percentages of CRs in either the acquisition experiment (*r* = 0.035, *P* = 0.460) or the savings experiment (*r* =−0.198, *P* = 0.279).

## Discussion

Three sessions of delay eyeblink conditioning were performed to assess acquisition, savings and extinction of a conditioned response (CR) of subjects with FRAXA and controls. Overall, subjects with FRAXA displayed fewer CRs than controls. In the acquisition experiment, the FRAXA group showed a slower increase in the percentage of CRs. In the savings experiment, both subjects with FRAXA and controls showed saving of the CR. Especially, the FRAXA group improved as the percentages of CRs no longer significantly differ from those of the controls. Extinction of the learned behavior in subjects with FRAXA was characterized by a swift decline in the percentage of CRs in the first trials. After that, the shape of the extinction curve was similar for subjects with FRAXA and controls. We observed no correlation between IQ and performance during delay eyeblink conditioning. These new findings imply a long-term influence of the lack of FMRP on extinction but not on savings of the delay conditioned eyeblink response.

### Acquisition of the CR

We confirmed the previously observed difference in delay eyeblink conditioning performance in the acquisition experiment (Koekkoek *et al.* 2005). This affirms the notion that individuals with FRAXA have impaired cerebellar motor learning. Compared with patients with hereditary ataxia and patients with lesions restricted to the cerebellum, the acquisition rate is higher in subjects with FRAXA (Bracha *et al.* 2000, Timmann *et al.* 2005). The reason is that the cerebellar circuit in subjects with FRAXA is intact in contrast to patients with cerebellar ataxia.

### Savings of the CR

In the savings experiment, the difference in motor learning behavior between the subjects with FRAXA and controls was not significant. Moreover, both the FRAXA group and the control group had retained their increased percentage of CRs at the beginning of the savings experiment. This suggests that a long-term memory effect occurred in the FRAXA group despite their impairments in CR acquisition. This new finding in humans seems to be at odds with repeated delay eyeblink conditioning experiments in global *FMR1* knockout mice. These mice did show improvement during repeated delay eyeblink experiments, although their percentage of CRs was always significantly lower than wild-type littermates (Koekkoek *et al.* 2005). Arguably no long-term savings effect was noticed in this study as the experiments were performed in 1 day, whereas in the present study the two experiments were separated by at least 6 months.

### Extinction of the CR

The rapid decrease in the number of CRs of individuals with FRAXA during the extinction experiment is remarkable. The fast extinction of the subjects with FRAXA in contrast to their slower acquisition implies that learning and unlearning of the CR are not direct opposites of the same mechanism. However, our results do not reveal whether extinction requires formation of a separate memory trace or extinction is a mask of the CR. Regardless of the exact mechanism for this type of motor learning, our results suggest that extinction is accelerated by the absence of FMRP.

### IQ of the subjects with FRAXA

The IQ scores, estimated by the Raven test, are high compared with those in other studies (Eliez *et al.* 2001; Fisch *et al.* 2007). However, we suspect that our subjects with FRAXA represent the high end of the spectrum of the population with FRAXA, because parents are more likely to enroll their child in a study when they deem them able to participate successfully, thereby probably inducing a bias for individuals with higher IQs. The IQ scores did not correlate with the delay eyeblink conditioning results, which is in line with other studies in retarded individuals (Ohlrich & Ross 1968). More elaborate IQ tests or larger group sizes could possibly change these outcomes.

### Anatomical implications

Our finding that the percentage of CRs was impaired during the acquisition but normal during savings suggests that there are at least two different sites at which learning occurs, one affected by absence of FMRP and another less affected by absence of FMRP. In the cerebellum, FMRP is expressed in the cytoplasm of all neurons and strongest in Purkinje cells in which FMRP is also localized in the nucleolus (Bakker *et al.* 2000; Tamanini *et al.* 1997). Purkinje cells can only be found in the cerebellar cortex. Many authors suggest that during delay eyeblink conditioning, plasticity occurs in both the cerebellar cortex and deep cerebellar nuclei (for reviews, see Attwell *et al.* 2002; Christian & Thompson 2003; De Zeeuw & Yeo 2005; Ito 2002). Both parallel fiber–Purkinje cell synapses in the cerebellar cortex and cerebellar nuclei are vital for acquisition of the CR (Chen *et al.* 1996; Koekkoek *et al.* 2003), whereas only the interpositus nucleus (a deep cerebellar nucleus) is thought to be essential for long-term savings of the CR (Christian & Thompson 2005). The results of the present study are in agreement with these ideas. Acquisition, dependent on FMRP-deprived Purkinje cells, appears impaired in subjects with FRAXA. During savings, subjects with FRAXA delay eyeblink conditioning resemble control subjects’ performance. Savings could be mediated by the interpositus nucleus, whose functioning is possibly less dependent on FMRP expression.

Contrary to normal performance in the savings experiment, subjects with FRAXA display abnormal extinction behavior. The anterior lobe of the cerebellum is necessary for extinction (Perrett & Mauk 1995). No anatomical abnormalities have been reported about this region in individuals with FRAXA.

### Behavioral implications

In the present study, we show differences as well as similarities between subjects with FRAXA and controls. We state that these differences are due to a learning defect and are not a consequence of an inability to perform the task correctly. The FRAXA phenotype is characterized by attention deficits, which make many tasks difficult to perform. However, eyeblink conditioning is independent of awareness (Clark & Squire 1998; Smith *et al.* 2005), so low attention cannot explain the differences we observe. In addition, subjects with FRAXA have no difficulty hearing the tone (as tested before the experiments) or responding to the air puff. Koekkoek *et al.* (2005) showed that although conditioned response amplitudes were affected in subjects with FRAXA, unconditioned response kinematics were normal. On the other hand, acute stress has been shown to impair learning in female rats but not in male rats (Bangasser & Shors 2004; Duncko *et al.* 2007; Shors 2001). Individuals with FRAXA are often hyperexcitable and their mental state, induced by a new environment, new people and performing new tasks, could influence their learning abilities. This might explain the large increase in the percentage of CRs during the savings experiment as they already know what is going to happen and are therefore likely to be more relaxed. Besides, in general, subjects appeared relaxed and involved in watching their favorite movie.

FRAXA is often associated with autism (Rogers *et al.* 2001). Several behavioral symptoms and cerebellar neuroanatomical abnormalities are found in individuals in both disorders (Belmonte & Carper 1998; Kaufmann *et al.* 2003; Rogers *et al.* 2001). During eyeblink conditioning, both subjects with autism (Sears *et al.* 1994) and FRAXA show faster extinction of the CR. However, subjects with autism also show faster acquisition of the CR contrary to the slower acquisition observed in the subjects with FRAXA. Thus, although both disorders appear to have a cerebellar component, at present one cannot pinpoint a common pathological mechanism underlying the symptoms.

### Clinical implications

To date, there is no drug available addressing or preventing the behavioral features of individuals with FRAXA. In a previous study, we described an individual with FRAXA who presented with exceptionally good cognitive, motor and behavioral capacities and had reached a high level of self-sufficiency (Govaerts *et al.* 2007). His performance might be the result of intensive mental and physical training he received throughout childhood. The acquisition experiment suggested that cerebellar plasticity is affected in patients with FRAXA, but our results also showed that a significant improvement in motor learning is achieved by repeating the same experiment. Therefore, including more physical exercise in training programs could be beneficial to compensate for the reduced cerebellar plasticity. An important challenge remains to further understand this disorder to be able to improve guidance and treatment of individuals with FRAXA.

## Conclusions

Different performance during acquisition and savings shows that the well-defined genetic mutation of FRAXA provides an interesting model to study the location of plasticity involved in delay eyeblink conditioning. We can conclude that absence of FMRP significantly influences cerebellar motor learning in individuals with FRAXA. On the long term, subjects with FRAXA can save and thereafter rapidly extinct the delay CR. Presence of savings and extinction behavior in FRAXA suggests that different sites or mechanisms of learning and storage of the memory traces are involved in these types of associative learning than in acquisition of the CR.
